# Blood Disease

**Published:** 1860-05

**Authors:** 


ART. III.—*Blood Disease.* By J. VAUGHAN HUGHES, M.D., late Acting Staff Surgeon to the British Forces in the Crimea, etc., etc. Churchill: London, 1859. pp. 132.

THE doctrine that a morbific matter in the blood is the immediate cause of many diseases, and that its concoction and elimination are necessary for their cure, is as old as the days of the Father of Medicine. In modern times this doctrine has received more definite statement and support from Liebig, and by calling to its aid so many of the phenomena of contagious and epidemic diseases, otherwise inexplicable, become clearly intelligible, that the *zymotic theory* has received countenance from the best medical writers, and is especially popular with the profession at large. The researches of modern pathologists, too, sustain the doctrines, without having yet succeeded in identifying the morbific agent itself, discovering its place of origin, or describing the cause of its development.

In the works of Paget, Simon, Virchow, and Williams, we find recorded a large amount of information upon diseased conditions of the blood; all, indeed, that we have yet succeeded in mastering. But from a study of these works we not only learn the present state of our knowledge, but also obtain an idea of the vast extent and great importance of that territory which yet lies beyond our ken, and which remains to be explored, mapped, and described by future investigators. We live in the firm belief that we shall see much of this territory conquered, and annexed to our present possessions; but at the same time feel assured that he who undertakes it has no holiday task before him. Indeed the probability is, that no one mind will ever discover and teach the great truths of this subject which now lie hidden from us, but that, like all great advances, it will be made gradually, step by step, one and another adding his mite, until the whole is known. Even if we are not obliged to wait for the assistance of a more perfect chemistry, we must have a large addition to our present knowledge of the composition of the various fluids of the body in disease—the analyses must be numerous and rigidly accurate—accompanying them must be carefully recorded observations of the symptoms which occurred at the same time, and when a sufficient number of these have been obtained, a candid and close examination must be made of the connection existing between the simultaneously occurring phenomena, and conclusions drawn which may afford a basis for practice. It is needless to say that this part of the task can only be committed to minds of a high order and free from a suspicion of bias or prejudice.

It is safe then to say, that whoever presents to the profession more accurate information upon the conditions of the blood in disease, or identifies certain series of morbid symptoms as depending upon abnormal conditions of the circulating fluid already known, lays the profession under obligations which, we are sure, will be acknowledged. Even less than this, would be a task worthy of commendation. To collect from the various sources all the facts recorded by different observers, to present them in a single treatise clearly and connectedly, to harmonize as far as possible what now appears conflicting, and compare what seems similar, and thus enable us to separate at a glance the known from the unknown, would be to confer a great benefit upon scientific medicine.

We regret to be obliged to say that neither of these tasks has been performed by the author, in the work under examination. We hoped to find in the book some additions to our present knowledge, and in the writer one who was laboring earnestly, although perchance humbly, in a field of scientific research so much needing cultivation. We have been disappointed in both respects. The book does not contain a single analysis of the blood, or of any fluid of the body, either original or the work of others; there are no cases recorded in which the symptoms are shown in connection with changes found in the circulating fluid; indeed, the author treats physiological and pathological chemistry almost as if they did not exist, and substitutes for them the crudest notions and the grossest mechanical views of the effects of deranged digestion. He attributes far more to "blood poison" than our present knowledge will justify, without presenting such clear notions of its nature and mode of action as would alone justify him in substituting this toxicological term for the simpler ones of gastric derangement, indigestion, or even the popular one, "bilious." We select a few paragraphs from the work, in illustration of the truth of our remarks:—

"Foremost * * * * as acting most frequently as a vitiating source, and reflecting its mischief on the brain and mind, may be regarded functional disorder of one or more of the chylopoietic viscera—that chain of organs constituting an apparatus which is concerned in the digestive or vegetative functions of life. And we may safely and undoubtedly conclude, if there be one organ above the rest in this chain which is more liable than the rest to render the blood impure, it is that huge secreting and depurating viscus, the liver."—pp. 3, 4.

"In temperate climes, and more particularly in England, where a large proportion of the population luxuriate in a heavy nitrogenous diet, * * * * attacks of functional derangment, varying in degree, occur in the liver; and this arises in consequence of the great mass of blood, which is formed by this kind of food, and propelled through its structure, creating a larger quantity of secretion than the organ can dispose of, or the wants of the system require. Hence an accumulation of bile takes place in the channels, together with the gall-bladder; and this collection cannot find a free exit into the bowel, because the main duct is plugged at its mouth with thick tenacious mucus, (!) another effect of this kind of diet to which we shall again refer."—p. 5.

"Should the acrid bile burst forth from its pent-up duct, and trickle down the surface of the membrane of the intestines, the irritation which it induces is so great that an unusual amount of mucous secretion is poured out over the internal lining of the bowel; this tenacious effusion, there accumulating and hardening, (!) forms a thick impervious artificial coating that acts indirectly, but most injuriously, on the general system, by obstructing the free absorption of nourishment from the intestines, and by obstinately counteracting the effects of ordinary aperients: hence a costive state is produced, which adds to the general distress, by inducing a plethoric condition of the abdominal blood-vessels, giving rise to hæmorrhoids or menorrhagia."—pp. 13, 14.

"Associated invariably with these evils, and bringing the gouty diathesis to a climax, we may always observe that the vegetative department of the frame rises to an overwhelming pitch (!) with a surplus of blood. Organs are distended and oppressed—their secretions increased and stagnating from want of application and disbursement, and while rendered inspissated by stagnation, they set up an irritation, which makes their respective organs hotbeds for brewing continued mischief, till, by absorption of this morbific mud, the circulating fluid becomes what has been already described, a reservoir of acrid and irritating impurities—a circle of floating evils," etc., etc.—p. 53.

That diseases are produced by morbid matters in the blood—that these matters have their origin in faulty digestion—that a cure is to be effected by eliminating these matters from the system—that the channels for elimination are the bowels, skin, and kidneys,—these are the doctrines of the book. But there is nothing new in all this, except the extent to which it is carried and the substitution of the meaningless term of

"blood poison," for more simple terms. It is all before the profession now; the student finds it in his text-books on physiology and the principles of medicine, and the practitioner in every work to which he refers. The chapter upon "Blood Disease in Relation to Gout" contains not nearly all of what is given by every writer upon the subject, and nothing in addition; the substance of the chapter upon "Blood Disease in its Relation to Affections of the Uterus" is in every work on diseases of females, under the head of Menorrhagia; and the whole work is but Mr. Abernethy's doctrine of the constitutional origin of local diseases, far more feebly presented than it was by that great and singular man. The subject is as familiar to the profession as "household words," and no respectable practitioner thinks of treating the local ailments of his patients without giving due attention to the state of the general system.

But when the treatise upon blood disease, which we hope some day to welcome, shall appear, we shall not only look for more clearness and exactness than characterizes the present one, but also for more correctness and elegance of language than is shown in the following paragraphs, to which we have added the italics:—

"The effects of blood disease on the brain and mind must be deemed the most important of any in the long category of bodily disorders which it can give rise to, and therefore demands in the first place more frequent allusion, and to be more deeply investigated, than any functional or organic mischief which may arise from the same cause in any less *dangerous* quarter of the frame."—p. 2.

"We cannot employ the surface of the mucous membrane of the intestines for this purpose, to the same extent that we can use the kidneys or skin—that is, in severe *rotten cases*, we cannot make the liver, pancreas, and bowel disgorge their corrupting contents by one or two forcible strokes," etc.—p. 26.

"Under such circumstances as these, hardly any case can prove so obstinate and deep rooted *but what* it would yield by perseverance in one persistent course of well-doing."—p. 57.

"Such a state tends to constipate the bowels, and to *bung up* the secretion of the liver and pancreas."—p. 64.

We ought not to find fault with this term "bung up," so much more expressive than euphonious, since it is in accordance with the mechanical views of secretion arrested by plugs of inspissated mucus impacted in the excretory duct of organs, which we have already given, and which the author repeats several times.

We must object also to such spelling as "*alkalie*" and "*stramony.*"

There is so much to censure in this small work, and so little to praise, that the question is forced upon us, why was it written? We think we can find an answer to the question in the general tone of the work; an answer which we receive with regret, but which the facts force upon us. The frequent paragraphs throughout the work evidently intended for readers below the professional level, the delicate hints of superior tact and discrimination possessed by *some* intelligent and well-read physicians, the occasional flings at the folly and absurdity of "drugging," so sure to please the taste of the lay reader, and the exaggeration which prevails whenever the effects of deranged digestion are described, leave no doubts in our mind as to which was the strongest inducement to write it, the needs of science or of the author. The public mind of England has lately been much exercised in regard to the confinement of some persons in lunatic asylums; within the first four pages of the work, we find insanity, as produced by the derangement of the stomach and bowels, so prominently presented that the non-professional reader would be sure to consider it a most frequent cause, and if troubled with slight indigestion would plainly see a lunatic asylum in the distance, to which he was surely hastening. Then again, deranged digestion, with all its train of real and imaginary evils, has ever been the subject to which the popular mind gives readiest attention, and the humoral pathology, in the broadest sense of the term, is the only doctrine of disease for the people. Putting all these things together, we find it impossible to escape the conviction already expressed; we again say that we regret to make so harsh a judgment public, but our duty to our readers requires that we should seek to guard them against deception by the attractive title of a work which certainly has no claims to scientific value.

